# Multifunctional Casein-Based Wound Dressing Capable of Monitoring and Moderating the Proteolytic Activity of Chronic Wounds

**DOI:** 10.1021/acs.biomac.3c00910

**Published:** 2024-01-31

**Authors:** Davood Kolahreez, Laleh Ghasemi-Mobarakeh, Felice Quartinello, Falk W. Liebner, Georg M. Guebitz, Doris Ribitsch

**Affiliations:** † Department of Textile Engineering, Isfahan University of Technology, Isfahan 84156-83111, Iran; ‡ Institute of Environmental Biotechnology, Department of Agrobiotechnology, IFA-Tulln, University of Natural Resources and Life Sciences, Vienna, Konrad-Lorenz-Strasse 20, 3430 Tulln an der Donau, Austria; § Institute of Chemistry of Renewable Resources, Department of Chemistry, University of Natural Resources and Life Sciences, Vienna, Konrad-Lorenz-Strasse 24, 3430 Tulln an der Donau, Austria; ∥ Austrian Centre of Industrial Biotechnology (ACIB), Konrad-Lorenz-Strasse 20, 3430 Tulln an der Donau, Austria

## Abstract

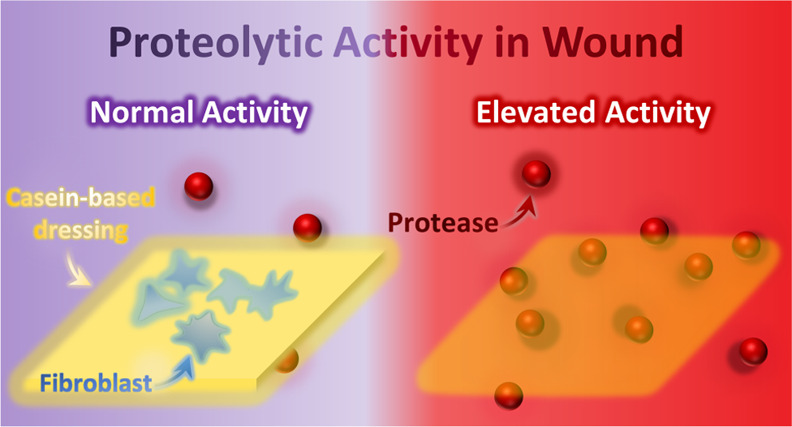

Every 1.2 s, a diabetic foot ulcer is developed, and every 20 s, one amputation is carried out in diabetic patients. Monitoring and controlling protease activity have been considered as a strategy for more efficient management of diabetic and other chronic wounds. This study aimed to develop a casein-based dressing that, by its disappearance, provides information about the activity of proteases and simultaneously harnesses proteolytic activity. Casein films were fabricated by using an aqueous solution, and heat treatment was successfully deployed as a green and clean approach to confer hydrolytic stability. Our results showed that casein-based films’ mechanical characteristics, water absorption, and proteolytic stability could be controlled by the length of the heat treatment, which proved to be a useful tool. An increase in the treatment duration from 30 min to 3 h led to toleration of 2.4 times higher stress, 2 times lower water uptake, and 3.4 times higher proteolytic stability at examined conditions. Selected casein-based structures responded to *Bacillus sp.* bacteria’s protease (BSP) and human neutrophil elastase (HNE) as representatives of bacterial and nonbacterial proteases found in the wounds at 10 and 200 ng mL^–1^ levels, respectively. The hydrolysis was accompanied by a 36% reduction in proteolytic activity measured by using a casein-based universal protease activity assay. The released casein fragments could scavenge 90% of the examined radicals. *In-vitro* cell culture studies showed that the hydrolysates were not cytotoxic, and the casein-based film had a favorable interaction with fibroblast cells, indicating its potential as a scaffold in the case that proteolytic activity would not be to the extent that causes its rapid disintegration. In general, these findings hold promise for applying the developed casein-based structure for detecting proteolytic activity without the need for any equipment, kits, or expertise and, more importantly, in a highly economical manner. In the case that the proteolytic activity would not be severe, it could also serve as a substrate for cell adhesion and growth; this would aid in the healing process.

## 1 Introduction

Chronic wounds do not pass the normal healing process, which arbitrarily consists of hemostasis, inflammation, proliferation, and remodeling phases. Various systemic and local issues, such as high age, immobility, nutritional deficiency, blood supply insufficiency, neuropathy, and infection, contribute to stagnant wounds.^1^ Pressure ulcers, diabetic foot, venous leg, and ischemic ulcers are four common types of chronic wounds. The microenvironment of these wounds could be distinguished from acute wounds due to the factors that some mentioned earlier; one distinguishing characteristic could be an abnormally high protease level. For instance, Kupczyk et al. compared the blood serum levels of some matrix metalloproteinases (MMPs) in healthy individuals and diabetic patients who had ulcers and found that MMP-2 and -3 were significantly higher in the diabetic group, while MMP-9 and -13 did not show any significant difference between the two groups.^2^ According to Chang and Nguyen’s findings, there was eight times as much active MMP-8 in human diabetes ulcers as there was in skin tissue from nondiabetic people.^3^ Mikosiński and colleagues reported that HNE and MM-2 concentrations were higher in wound fluids from venous leg ulcers than those obtained from donor sites in split skin grafting.^4^ It is difficult to pinpoint the exact mechanisms that lead to elevated protease expression or activity, but it seems that some factors, such as diabetes,^2^ ischemia,^5^ high age,^6^ and bacteria,^7^ could be influential.

Given the destructive role of abnormally high proteolytic activity, a group of experts suggested that controlling and monitoring the protease activity could be beneficial for providing a more efficient treatment.^8^ An advanced and expensive therapy such as tissue grafting could fail in part due to the noxious protease activity.^9^ In our previous study, gelatin beads were cross-linked with glycerol diglycidyl ether and dyed with one reactive dye.^10^ The cleavage of gelatin by proteases was accompanied by the release of colored fragments. Moreover, a striking difference in the color of fluids from infected and uninfected wounds was noted.^10^ Hasmann et al. bonded a reactive dye (Remazol brilliant blue) to peptidoglycan and then embedded it in an agarose matrix or alginate beads.^11^ The release of dye-conjugated fragments from beads correlated with the lysozyme or protease activity and the condition of wound fluid in relation to the bacterial load. In another study in our group, chromogenic peptide substrates of HNE and cathepsin G were conjugated to various substrates, including silica gel, and again, a distinction was recognized between the color intensity of hydrolysates produced upon exposure to the infected and noninfected wounds’ fluids.^12^ Furthermore, the potential of cellulosic and nanocellulosic substrates for immobilizing HNE-sensitive fluorogenic peptides was explored.^13^ The color change concept has been pursued in many other studies, and usually, only one or a maximum of two proteases have been targeted.^14^

There are different approaches for managing the proteases’ activity. They could be removed, at least partially, along with fluids that are absorbed into the dressing.^15^ The second way is to mitigate their activity, for example, by incorporating an inhibitor into the dressing.^16−18^ Another strategy in dealing with protease issues is control of their expression at the gene and protein levels; for instance, tannic acid/siRNA nanogels were prepared and embedded in a hydrogel matrix. The results showed that reactive oxygen species cause matrix breakage and release of nanogels.^19^ It has been claimed that tannic acid is hydrolyzed when macrophages internalize nanoparticles, and as a result, siRNA releases and partially silences the MMP-9 genes.

Casein constitutes approximately 80% of bovine milk proteins. Acidic casein is obtained through precipitation from milk by adjusting the pH to the casein isoelectric point (4.6) by using inorganic acids. Neutralization of acid casein with sodium hydroxide leads to the formation of sodium caseinate, which is water-soluble. Casein has been studied for use in a number of applications, including controlled release systems,^20^ textile fibers,^21^ flame-retardant compounds,^22^ and edible films.^23^ In the biomaterials area, its mixture with polycaprolactone (PCL) and gelatin for the fabrication of electrospun webs and application in cartilage tissue engineering has been considered.^24^ In another research, scaffolds made of reduced-graphene oxide/polypyrrole were coated with casein phosphopeptide, which had been concomitant with a greater proliferation of osteoblast-like cells.^25^ A recent study reported the preparation of some structures with antibacterial and blood-clotting properties by simultaneous electrospinning of casein/poly(vinyl alcohol) (PVA) and electrospraying of casein solutions containing a metal–organic framework.^26^ Another recent study reported that treating wounds with PCL/casein fiber constructs resulted in more granulation tissue and lower levels of proinflammatory cytokines than treating wounds with similar structures made of PCL alone.^27^ Zhu and colleagues developed a photocurable casein-based hydrogel with the perspective application in emergency wounds.^28^ The susceptibility of casein to proteolysis has been exploited in protease assays^29^ and for developing systems that respond to bacterial proteases^30^ or the proteolytic milieu of tumor tissues.^31^

The present research aimed to develop a casein-based substrate that responds to a variety of proteases offers insight into the overall proteolytic activity rather than just the activity of one or two specific proteases, and does this without depending on a special instrument, kit, or even skill. The casein-based dressing was fabricated through a clean and cost-effective approach. The response of the fabricated structure to various proteases and changes in proteolytic activity was investigated. *In-vitro* tests were also carried out to assess the cytocompatibility of hydrolysates and cell–biomaterial interactions.

## 2 Experimental Section

### 2.1 Materials

Casein sodium salt from bovine milk (C8654), acetic acid, hydrindantin, ninhydrin, l-tyrosine, trichloroacetic acid, sodium carbonate, Folin–Ciocalteu′s reagent, ethanol, bovine serum albumin, 3-(4,5-dimethylthiazol-2-yl)-2,5-diphenyltetrazolium bromide (MTT), Mueller-Hinton agar, and dimethyl sulfoxide (DMSO, only for MTT assay) were purchased from Sigma-Aldrich (Steinheim, Germany). BSP (P5985) was supplied by Sigma-Aldrich (Copenhagen, Denmark). Phosphate-buffered saline (PBS) for preparing protease solutions or dilutions was obtained from Sigma-Aldrich (St. Louis). Roti Quant (Bradford reagent), DMSO for the ninhydrin assay, sodium acetate trihydrate, and glycerol were bought from Carl Roth GmbH (Karlsruhe, Germany). Glycine was from Merck (Darmstadt, Germany), ammonium persulfate (APS) was obtained from Fluka (Buch, Austria), and 2,2′-azino-bis(3-ethylbenzothiazoline-6-sulfonic acid) (ABTS) was provided by Roche (Mannheim, Germany). Porcine pancreatic elastase (PPE) with a specific activity of 2.22 U μg^–1^ was supplied by Abnova (Taipei City, Taiwan). HNE with a specific activity of 6.04 U μg^–1^ was obtained from Aviva Systems Biology (San Diego). Glutaraldehyde was obtained from Junsei (Tokyo, Japan). Collagenase type I, PBS for the cell attachment test, fetal bovine serum (FBS), Roswell Park Memorial Institute (RPMI) 1640, and Dulbecco’s Modified Eagle’s Medium (DMEM) were provided by Bioidea (Tehran, Iran). PBS for the MTT assay was supplied by Nanofanavar Alaa (Isfahan, Iran).

### 2.2 Methods

#### 2.2.1 Fabrication of Casein Films

Sodium caseinate aqueous solutions (12 wt %), containing glycerol 50% of the casein mass, were prepared by stirring and heating the mixture at boiling temperature for 30 min. Sodium caseinate will be mentioned as casein in the rest of the text. Then, the solution was cast on a poly(tetrafluoroethylene) (PTFE) sheet and spread evenly. The as-prepared films were heat-treated in a conventional oven at 150 °C for 30, 60, or 180 min (the relative humidity inside the oven was not regulated).

#### 2.2.2 Attenuated Total Reflectance Fourier-Transform Infrared Spectroscopy (ATR-FTIR)

ATR-FTIR analysis was carried out using a PerkinElmer Spectrum 100 FTIR Spectrometer (Shelton). Measurements were performed covering the vibrational range of 600 to 4000 cm^–1^ at a resolution of 4 cm^–1^, averaging 16 scans for each sample.

#### 2.2.3 X-ray Diffraction (XRD) Analysis

The wide-angle X-ray diffraction (WAXD) patterns of the casein-based films were obtained by using a Philips PW1730 X-ray diffractometer (Amsterdam, Netherlands). The angular range was 2Θ = 5–60° with an increment of 0.05°. Data were collected for 1 s at each angle. Cu–Kα radiation (λ = 1.54056 Å) was produced by an X-ray source at 40 kV and 30 mA.

#### 2.2.4 Differential Scanning Calorimetry (DSC)

The thermal behavior of casein-based films (with and without glycerol) was investigated using a NETZSCH DSC 214 Polyma differential scanning calorimeter (Selb, Germany). The samples were heated from 25 to 400 °C at a rate of 10 °C min^–1^, and the DSC thermographs were recorded.

#### 2.2.5 Field Emission Scanning Electron Microscopy (FE-SEM)

The samples were imaged using an FEI Quanta FEG 450 (Oregon) FE-SEM. They were coated with gold using an Agar auto sputter coater 108A (Essex, U.K.) for either 200 or 250 s (20 mA) before imaging.

#### 2.2.6 Tensile Mechanical Tests

Specimens with rectangular shapes (30 × 5 mm) were cut from the larger films. Their thicknesses were measured by using a digital micrometer (Schut Geometrical Metrology GmbH, Trossingen, Germany). Tests were conducted using a Karl Frank GmbH (Weinheim, Germany) type 81559 tensile testing machine equipped with Zwick Roell (Ulm, Germany) software. All tests were performed at a constant elongation rate adjusted to 10 mm min^–1^, a preload of 50 kPa, and a gauge length of 2 cm.

#### 2.2.7 Contact Angle

The static contact angle was measured by depositing a 2 μL of deionized water droplet on the sample and measuring the angle formed by the droplet and the surface at different time points (CA-500A, Sharif Solar, Tehran, Iran). The dynamic water contact angle was evaluated by using a Dataphysics DCAT 11 tensiometer (Filderstadt, Germany). To this end, rectangular samples (30 mm × 5 mm) were used, the immersion depth was 5 mm (0.2 mm S^1–^), and the average of the samples’ thicknesses was also entered into the software.

#### 2.2.8 Water Absorption

Specimens (10 mm × 10 mm) that were heat treated for 30 min, 1 h, and 3 h at 150 °C were incubated in PBS for 24 h, of which 22 and 45 min were at 37 °C. The ratio of the PBS solution volume (mL) to the sample’s mass (mg) was 0.1. The samples’ initial mass (*M*_i_) was measured before they were immersed in PBS. The wet mass (*M*_w_) was measured after removing excess water from the samples’ surfaces at the end of incubation. After the mass of wet samples was measured, they were dried, and their mass was measured (*M*_f_). The water absorption was calculated according to the following formulas.
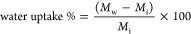
1
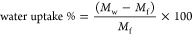
2

#### 2.2.9 Study of Sample Stability in Proteolytic and Nonproteolytic Mediums

In order to compare the stability of samples made of casein alone (without additive) and those that contained additive (glycerol), heat-treated (150 °C for 1 h), and nonheat-treated, they were incubated in PBS and PBS containing BSP (10 ng mL^–1^). The examined specimens’ thickness ranged from 46 to 53 μm, measured with a Schut Geometrical Metrology digital micrometer (Trossingen, Germany). The ratio of solution volume (mL) to theoretical casein mass (mg) in the samples was 0.15. The theoretical casein mass was assumed to be equal to the samples’ mass for specimens made of casein alone and 2/3 of the samples’ mass for additive-containing samples. This was because the additive content was 50% of the casein mass in the solutions used in the casting step, and therefore, the theoretical content of the additive should be 1/3 of the final sample mass. Incubation was conducted in a shaker incubator at 37 °C, applying a shaking frequency of 150 rpm. After 20 h, the specimens’ deterioration state was appraised either qualitatively by visual inspection (documented by taking pictures) or quantitatively by quantification of the primary amines of species released into the medium (cf. Section 2.2.10).

Glycerol-containing casein samples (10 × 10 mm) that were heat treated for 30 minutes (30-min) or 3 hours (3-h) were incubated in PBS containing various BSP levels in a 24-well plate. Selected samples (30-min) were also incubated in PBS containing different concentrations of HNE or PPE. Smaller-scale incubations (3.0 ± 0.2 mg, 30-min samples, 96-well plate) with PPE (20 g mL^–1^) and BSP (10 g mL^–1^) for 1 and 5 days were carried out to assess the potential change in proteolytic activity upon hydrolysis of the specimens. PPE is a model for human leukocyte elastase,^32^ a key enzyme in the inflammatory response. The rationale behind choosing PPE and BSP concentrations for this part was to meet the detection limit of the activity assay (Section 2.2.11). The 30-min heat-treated samples were subjected to PBS containing collagenase type I (500 μg mL^–1^) for 1 day in order to evaluate the hydrolysates’ antibacterial and cytocompatibility (cf. Sections 2.2.13 and 2.2.14.2, respectively). The hydrolysates were heated for 10 min at 90 ± 3 °C to deactivate the protease before being deployed in subsequent experiments. A control group without casein-based samples (solutions alone) was included in the experiments and incubated under the same conditions as the test groups. The specimen thickness for all sets ranged from 60 to 80 μm. The ratio between the volume of solution (mL) and the samples’ mass (mg) was 0.1. All incubations were conducted in a shaker incubator with a 37 °C temperature and a 150 rpm shaking rate.

#### 2.2.10 Quantification of Primary Amines

Photometric quantification of water-soluble protein fragments, employing the well-known color reaction of ninhydrin with primary amine groups abundant on the outside surfaces of native protein tertiary structures at physiological conditions and the *N*-terminus of each polypeptide chain, was conducted to quantify the samples’ hydrolysis. The assay was carried out according to the protocol used by Quartinello et al.^33^

#### 2.2.11 Protease Activity Assay

This test was conducted similarly to a previously published protocol^29^ with minor changes; especially, the volumes were adjusted to be compatible with performing the test on a smaller scale. A 0.65 wt % sodium caseinate solution in PBS was prepared. Aliquots of this solution (100 μL) were transferred into 1.5 mL microtubes. Then, 20 μL of each sample was added, and after homogenization, the mixtures were incubated at 37 °C for 10 min. Subsequently, 100 μL of 110 mM trichloroacetic acid was added. After incubating at 37 °C for 30 min and centrifuging for 15 min, 200 μL of supernatant from each of the test solutions was transferred to a 1.5 mL microtube. Then, 500 μL of sodium carbonate (0.5 M) and 100 μL of Folin-Ciocalteu’s reagent (2 N) were added. After short-term vortexing, all samples were incubated at 37 °C for 30 min. Finally, 200 μL of each sample was pipetted in triplicate into a 96-well plate. Absorbance was measured at 760 nm by using a Tecan Infinite 200 Pro microplate reader (Männedorf, Switzerland). l-tyrosine solutions in Milli-Q water were used for the preparation of the calibration curve; the experimental process was similar to the last part of the assay mentioned above.

#### 2.2.12 Antioxidant Test

The test was performed according to ref (34). The decolorization percentage was calculated based on the following eq (10 μL of PBS was used instead of analyte for control; as the blank, 10 μL of PBS plus 190 μL of Milli-Q water was used):
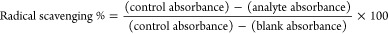


#### 2.2.13 Antibacterial Test

The sensitivity of *Staphylococcus aureus* ATCC 6538 and *Escherichia coli* ATCC 10536 to hydrolysates was evaluated using the disc diffusion method according to standard number 13560 of the Institute of Standards and Industrial Research of Iran (ISIRI). Tests were carried out for 24 h. In each well, 40 μL of the sample was deployed.

#### 2.2.14 *In-Vitro* Experiments

##### 2.2.14.1 Cell–Biomaterial Interaction

Before cell seeding, each side of casein-based films was exposed to UV light for 1 h. Mouse fibroblasts (L929) with a density of 10^5^ in DMEM medium supplemented with FBS (10% v/v) were seeded on samples and incubated for up to 7 days at 37 °C, 90% humidity, and 5% CO_2_. On days 5 and 7, samples were fixed by 2.5% (v/v) of glutaraldehyde for 1 h. After that, they were rinsed twice with PBS and subsequently dehydrated with different concentrations of ethanol, 25, 50, 75, and 96%, in each one for 15 min. Cell–biomaterial interaction was evaluated by taking FE-SEM images, as described in Section 2.2.5.

##### 2.2.14.2 Cytotoxicity Evaluation of Hydrolysates

The MTT assay was used to assess the cytocompatibility of the hydrolysates. Samples (hydrolysates and collagenase solution) were mixed with DMEM culture medium supplied with FBS (10% (v/v)) in a 30:70 ratio. L929 fibroblasts in DMEM medium containing 10% FBS were cultured in a 96-well plate (100 μL of the cell suspension with a cell density of 1.5 × 10^5^ cells mL^–1^ in each well). The next day, the media were aspirated and replaced with the specimen-containing media and fresh medium as the positive control (in triplicates). Two time intervals of 1 and 3 days were considered in these trials. On the day of the assay, the contents of the wells were aspirated, and 110 μL of a solution made of a mixture of colorless DMEM medium and MTT solution (5 mg mL^–1^ in PBS) in a 10:1 ratio was added to each well. After 4 h of incubation (37 °C, 90% humidity, and 5% CO_2_), 85 μL of the wells’ content was withdrawn, and 100 μL of DMSO was added to each well. Pipetting was done several times to ensure that the formazan crystals had dissolved. The absorbance was read at 570 nm upon shaking the plate in an enzyme-linked immunosorbent assay reader (BioTek ELx808, Winooski) equipped with Gen5 3.03 software.

#### 2.2.15 Statistical Analysis

Statistical analysis was performed using the two-sample *t*-test method at a significance level of 0.05, using the Origin 19 software package (Northampton). The number of replicates has been reported in each section separately, and results were reported as mean ± standard deviation (SD).

## 3 Results and Discussion

### 3.1 Discriminating the Heat and Additive Role and Insights into their Functions in Stabilization

**Figure 1:**
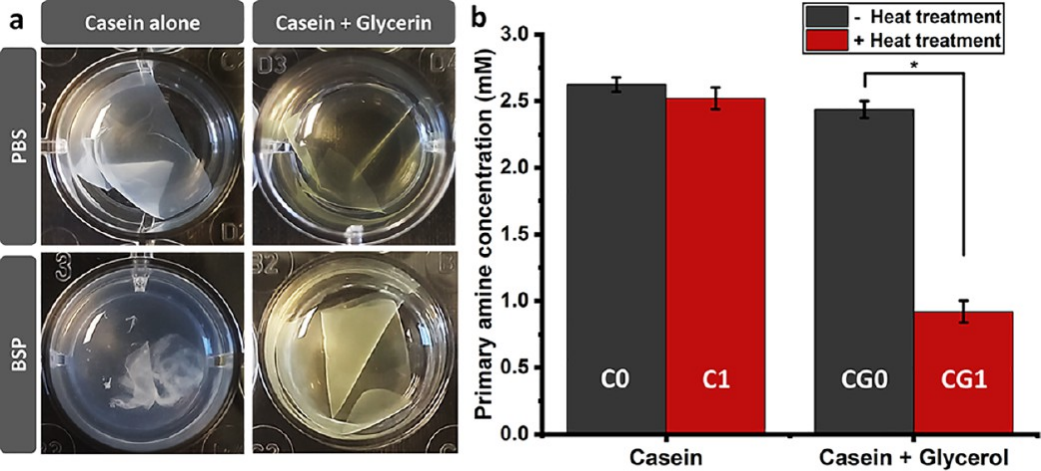
(a) Images of the heat-treated samples with and without glycerol after 20 h of incubation in PBS and PBS containing BSP (10 ng mL^–1^); (b) The primary amine concentration as a criterion for the hydrolysis degree in the supernatants obtained from incubation of samples in PBS containing BSP (10 ng mL^–1^). Data are the average of at least 4 measurements.

In the course of experiments for the cross-linking of casein-based structures, it became evident that heat treatment under certain conditions leads to hydrolytic stability. While there are a few relevant studies concerning casein heat treatment,^35−38^ they have some shortcomings. One point that needs to be clarified is distinguishing the role of heat from additives. Previous studies have not acknowledged the possibility that additives might have contributed to the stabilization in addition to their plasticizing effect. For this part of the research, 2 groups of specimens were prepared: one without additive and the other with glycerol; C0 and CG0, respectively, indicated that these samples were in a nonheat-treated state. Samples of these 2 groups that underwent 1 h of heat treatment at 150 °C have been marked as C1 and CG1, respectively. The hydrolytic stability of samples in PBS and PBS with BSP (10 ng mL^–1^) was evaluated after 20 h of incubation (37 °C, 150 rpm). As is evident in Figure 1a, in PBS, both C1 and CG1 retained their integrity, which shows that heat treatment at this condition has given rise to stabilization, even in the absence of glycerol; C0 and CG0 dissolve in both conditions (data not shown). It is concluded from this observation that the heat treatment of pure casein can positively affect its hydrolytic stability. Mecham and Olcott treated various proteins, including casein, in boiling hydrocarbons with boiling points of 110 to 203 °C for 18 h.^39^ Heat treatment at a certain temperature range, which differed for different proteins, had been associated with a reduction in the solubility in the investigated media. In the case of casein, solubility in phosphate buffer (pH around 10.7) was minimal for samples treated at 153 °C (boiling isopropylbenzene) compared to the lower and higher temperatures.^39^

On the other hand, in BSP, C1 samples lost their integrity after 20 h (Figure 1a), whereas CG1 did not. The primary amine content, as a criterion for the degree of hydrolysis, consistent with visual evaluations, confirmed that degradation was significantly more severe in the case of C1 compared to that of CG1 (Figure 1b). Furthermore, in contrast to the glycerol-containing group, where the primary amine content in the supernatant of CG0 was much higher than in CG1, the group that was without glycerol showed no significant difference in this regard between the C1 and C0 specimens (Figure 1b). These data could imply that glycerol has affected the effectiveness of heat treatment for stabilization; this point has been ignored in previous studies.^35−38^

**Figure 2:**
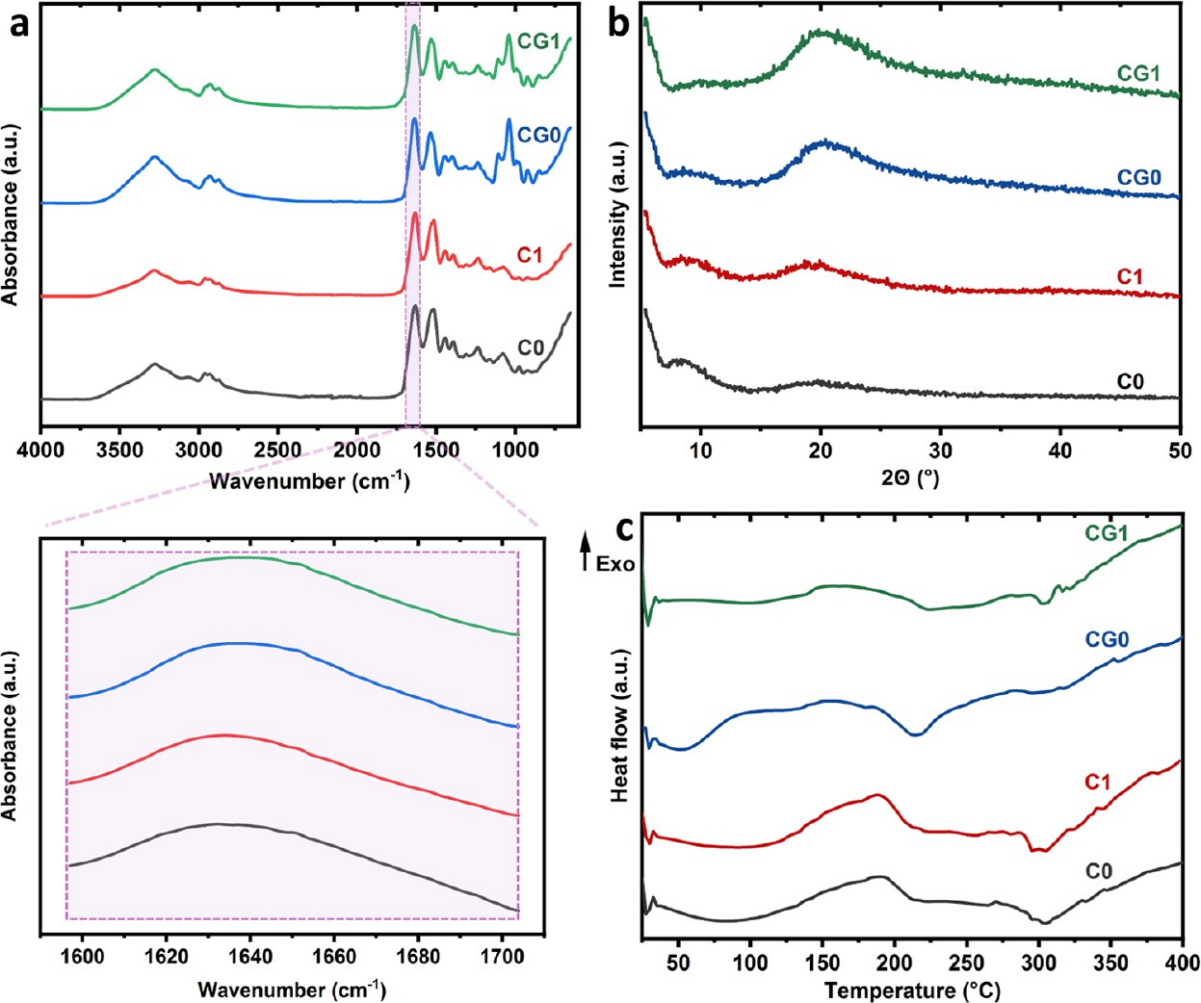
(a) ATR-FTIR spectra of the samples in the whole range and amide I region, (b) WAXD spectra, and (c) DSC thermographs. The samples were labeled as follows: C0 (no glycerol, no heat treatment), C1 (no glycerol, heat treated for 1 h), CG0 (with glycerol, no heat treatment), and CG1 (with glycerol, heat treated for 1 h).

No striking difference was recognized between the ATR-FTIR spectra of the samples that were not heat-treated, C0 and CG0, and heat-treated counterparts, C1 and CG1, respectively (Figure 2a). This observation does not support the hypothesis that new bonds have been formed, different from those already present. The only minor detected difference is a decrease in the peak height at 1040 cm^–1^ after heat treatment. This peak should be due to the stretching vibration of C–O in glycerol. One possible explanation for this change is that a portion of glycerol (intact or fragmented) might have left the sample during the heat treatment, as suggested by the headspace gas chromatography–mass spectroscopy analysis (data not shown). These observations substantiate the hypothesis that the plasticizing effect might be responsible for the higher efficacy of heat treatment in stabilizing the glycerol-containing structures.

The question is through which pathway heating causes the casein hydrolytic stability. The happenings could be physical, chemical, or both. As stated above, the FTIR spectra do not provide any conclusive evidence for the formation of new bonds that would not be already in the samples (Figure 2a). However, Mecham and Olcott’s study lends credence to the idea that functional groups of casein might react with each other. As mentioned above, they treated various proteins, including casein, in boiling hydrocarbons with boiling points of 110 to 203 °C for 18 h.^39^ It has been claimed that the group treated at 203 °C had fewer amino groups than the untreated group, as measured by the amount of ammonia produced.^39^ The numbers of the basic and acidic groups were also calculated by measuring the unbounded dye concentration.^39^ Compared to the control (untreated group), they were lower in the casein group heated at 182 °C. In another study, wool keratin was heated at temperatures in the range of 60 to 140 °C for 48 h; increasing in heat treatment temperature had been concurrent with a decrease in the number of free lysine side groups and an increase in the content of aspartyl-lysine and glutamyl-lysine isopeptides.^40^ In addition, heating at temperatures higher than 140 °C brought forth resistance to enzymatic hydrolysis.^40^ Nevertheless, further investigations are required to gain more insight into chemical reactions possibly involved in stabilizing casein as a result of heat treatment.

Physical events that could potentially lead to hydrolytic stability are crystal formation and changes in the secondary structure. In order to evaluate the change in the secondary structure of casein following heat treatment, the amide I region of the FTIR spectra was evaluated separately. The amide I zone in the FTIR spectrum (1597–1704 cm^–1^), which is mainly due to C=O vibrations, is known as an area that is sensitive to the secondary structure of proteins.^41^ There was no discernible difference between the FTIR spectra of C0, C1, CG0, and CG1 in this region (Figure 2a). Consistent with FTIR results, heat treatment has not led to the appearance of any crystalline peak in XRD spectra (Figure 2b). This is in agreement with previous studies reporting an amorphous XRD pattern for casein.^42^ The only features in the XRD patterns are two broad peaks around 8 and 20°, which have also been observed in earlier works.^43^ DSC analysis was also performed to obtain additional information about possible changes in the secondary structure, especially crystal formation, as a consequence of heat treatment. No strong signal was found in the thermographs that indicates the formation of crystals due to heat treatment (Figure 2c). It was expected that if, during the heat treatment, crystals were formed, endothermic peaks due to the melting of those crystals would appear in the thermograms of heat-treated samples (C1 and CG1), or if cold crystallization happens, it would show itself as exothermic peaks. In the case of CG1, an endothermic peak has appeared around 304 °C, which does not exist in CG0, but the point is that a peak also exists at the same temperature in C0 that was not heat treated; therefore, we cannot be sure that the aforementioned peak is due to the melting of crystals that have been formed during heat treatment. The endothermic peaks around 300 °C could be due to the breakage of some bonds in casein.^44^ According to Liu and colleagues’ report, based on differential thermogravimetry, the decomposition of casein was initiated at 291 °C and occurred at the highest rate at 317 °C.^45^ The broad peak at approximately 214 °C in the CG0 group could be due to glycerol evaporation; according to Almazrouei and colleagues’ study, around 97% of pure glycerol has been evaporated in the temperature range of 144–262 °C.^46^ The findings from ATR-FTIR, XRD, and DSC analysis do not support the idea that casein’s secondary structure might be affected by heat treatment, which is consistent with the general assumption that caseins are disordered molecules.^47^

### 3.2 Influence of Heat Treatment on Morphology

**Figure 3:**
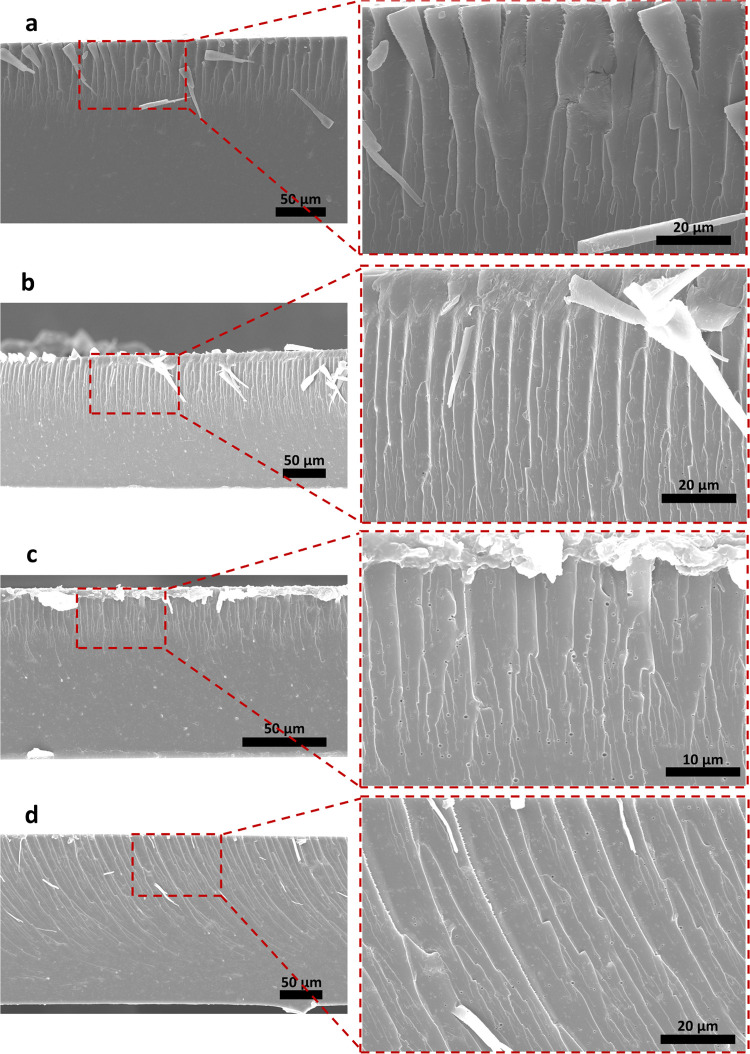
FE-SEM images of the fractured cross-section of the glycerol-containing sample that was not heat treated (a), heat treated for 30 min (b), 1 h (c), and 3 h (d), at 2 magnifications.

The fractured cross-section of the unheated sample has two distinct areas (Figure 3a); the region near the free surface that was exposed to air after casting seems different from the lower areas. The distinction is also recognizable in the cross-section of the samples heat-treated for different durations (Figure 3b–d). Interestingly, the cross-sectional morphology changed when the unheated sample was immersed in liquid nitrogen before breaking (Figure S1). The noticeable features near the surface in the samples broken without liquid nitrogen became less prominent in the samples broken after liquid nitrogen exposure. It is important to consider that the unusual features that appear near the upper surface may be artifacts of the sample-breaking process and may not exist in the intact samples. The heat-treated samples apparently have more pores than the unheated samples. This may result from the evaporation of water or glycerol from the samples in the early stages of the heat treatment. To our knowledge, the morphology of similar heat-treated casein-based structures has not been investigated in previous works to compare the results. However, the morphology of nonheat-treated samples that were broken following immersion in liquid nitrogen looks similar to the morphology observed in Aliheidari et al.’s study; they also used liquid nitrogen to break the specimens.^48^

### 3.3 Influence of Heat Treatment on Mechanical Characteristics

The mechanical properties of glycerol-containing films heat treated for 30 minutes (30-min), 1 hour (1-h), and 3 hours (3-h) were measured in tensile mode (Table 1). The nonheated samples lack hydrolytic stability, deform easily, even in exposure to the hands’ moisture, and are of no commonsense value, so they were not tested. Maximum tolerated stress and elastic modulus have increased as a function of heat treatment duration, while strain at maximum stress has been less affected. The strength and modulus of 3-h samples are approximately twice that of their 30-min heat-treated counterparts, indicating the high capacity of heat treatment for tuning the mechanical properties.

**Table 1:** Mechanical Properties of Films Heat-Treated for Different Durations^a^

	max. stress (MPa)	strain at max. stress (%)	elastic modulus (MPa)
30-min	13.44 ± 2.12	4.44 ± 0.34	6.45 ± 0.26
1-h	21.90 ± 3.04	4.47 ± 0.35	9.81 ± 0.27
3-h	32.52 ± 2.05	5.13 ± 0.18	12.63 ± 0.80

a All differences between groups were statistically significant (at 0.05 level), except for strain at maximum stress between the 30-min and 1-h groups. Data are the average of 4 measurements.

### 3.4 Effect of Heat Treatment on Hydrophilicity

**Figure 4:**
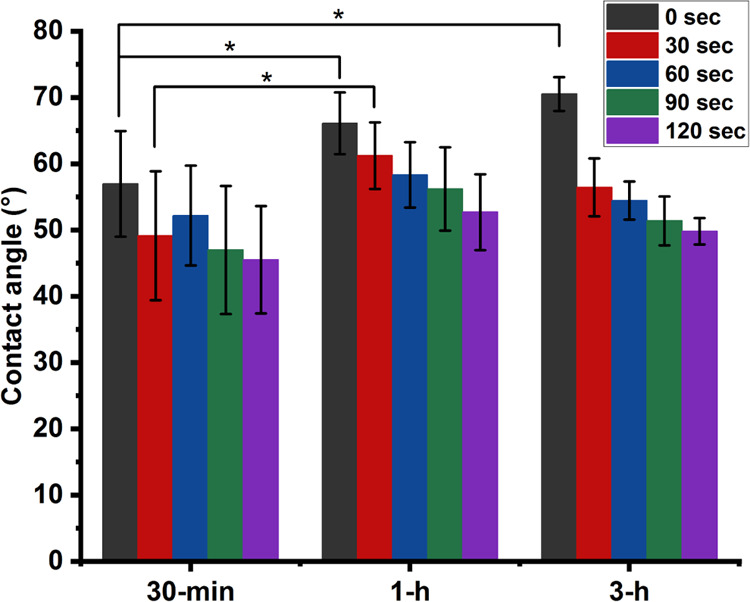
Static water contact angles were measured at different time points after depositing the water droplet over the glycerol-containing specimens heat-treated for 30 minutes (30-min), 1 hour (1-h), and 3 hours (3-h) at 150 °C. Data are the average of at least 3 measurements.

The surface hydrophilicity of specimens heat treated for different durations was compared by measuring the static water contact angle (SWCA). As shown in Figure 4, there were no significant differences between the 3 groups in most cases. Right after depositing the droplet over the samples, the SWCA was 70 ± 3° for the samples heat-treated for 3 h and 57 ± 8° for those treated for 30 min, indicating a higher hydrophobicity for the former. The only significant difference at the 30 s time-point after depositing the droplet was between the samples heat-treated for 30 min (49 ± 10°) and those treated for 1 h (61 ± 5°), with the former having a lower SWCA. The casein-based structures in this study are hydrophilic, as their SWCA is below 90°. No similar research with heat-treated casein samples was found to compare with the results obtained in the current study. However, some studies reported the SWCA of glycerol-containing casein films without heat treatment. Yin et al. measured an SWCA of about 63° (right after the droplet was placed on the surface) for casein films with a similar glycerol ratio as in current research.^49^ Another study reported an SWCA of 55° for casein films with 20% dry mass glycerol made by extrusion.^50^

Dynamic contact angle measurement was also performed. Different position intervals were considered for calculating the advancing and receding contact angles in the software associated with the instrument. In none of the investigated position ranges, the regression coefficient (RQ) that indicates the reliability of the calculated contact angles was near 1 for advancing contact angles (the calculated contact angles whose RQ is close to 1 are reliable according to the manufacturer’s instruction). For the position range of 4–5 mm, the RQ was more than 0.99 for the calculated receding contact angles for all replicates of all 3 groups (30-min, 1-h, and 3-h). For all samples, the obtained receding contact angle was zero. In addition to the water contact angle, the samples’ water uptake was also measured. The water uptake can illuminate the differences between the samples better than water contact angles because, in the case of static contact angle, only the hydrophilicity of the samples’ surfaces is evaluated, and the dynamic contact angle has some limitations regarding our specimens. For instance, exposing only a small part of the samples (5 mm) to the water, their swelling, insufficiency of the exposure time for reaching equilibrium water absorption, and release of some substances from the samples could compromise the capability of this method for revealing the differences between the samples in the current research.

**Figure 5:**
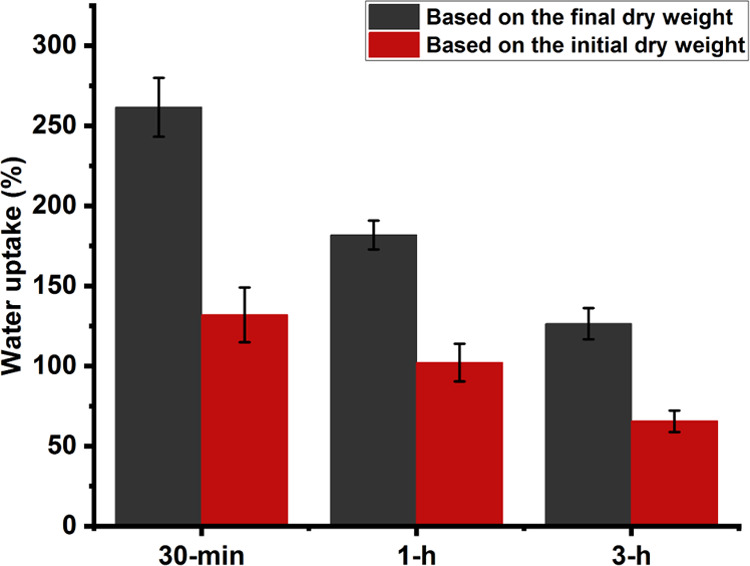
Water uptake (absorption) of the glycerol-containing samples that were heat treated for 30 minutes (30-min), 1 hour (1-h), and 3 hours (3-h) was calculated by considering the initial and final dry weight (24 h incubation in PBS). Regardless of the formula used, the water uptake values showed significant differences among the three groups of samples. Data are the average of at least 5 measurements.

Two methods were used to calculate the water uptake. The first method was the conventional one, which used the initial dry weight of the sample (eq 1).^51^ The water uptake obtained through this method varied from 65 ± 7% for the samples heated for 3 h to 132 ± 17% for those treated for 30 min (Figure 5). However, we believe that this approach is not the best choice for cases where the specimens lose some weight during the test and could cause a deviation from the actual water absorption capacity of the material and an underestimation of it. Therefore, in the current research, the water uptake was also calculated by inserting the final dry weight in the formula instead of the initial dry weight (eq 2). This calculation approach also revealed a significant difference in water absorption among samples treated for different durations. The water uptake spanned from 261 ± 18% for specimens heat-treated for 30 min to 126 ± 10% for those heated for 3 h (Figure 5). Moreover, this method gave much higher water uptake values for each group of samples, which were 77 to 98% higher than the first method. This shows that the weight loss during the test significantly affected the water uptake calculations. These results are novel and, to our knowledge, have not been reported in any previous work, so there is no similar study for comparison. The most relevant study is by Ghosh et al., who prepared a casein film without additives and heat-treated it for 18 h at 130 °C.^37^ They reported that the sample absorbed around 280% water (measured based on the conventional method mentioned before) in a few hours (3–4 h) but ultimately disintegrated.

The reduction in water uptake as the heat treatment duration increased could be attributed to physical and chemical changes in the samples. Physically, the heat treatment could make the structures more compact and reduce the free spaces that can hold water. Chemically, heat treatment could decrease the number of polar groups in the samples, which are responsible for attracting water molecules. Both of these effects can lower the water absorption capacity of the samples.

Although the primary indication for the use of structures developed in the present research is not moisture management, the findings presented above suggest that they may also contribute in this regard and absorb a portion of wound exudates.

### 3.5 Effect of Heat Treatment on the Sensitivity of Casein-Based Structures to BSP

**Figure 6:**
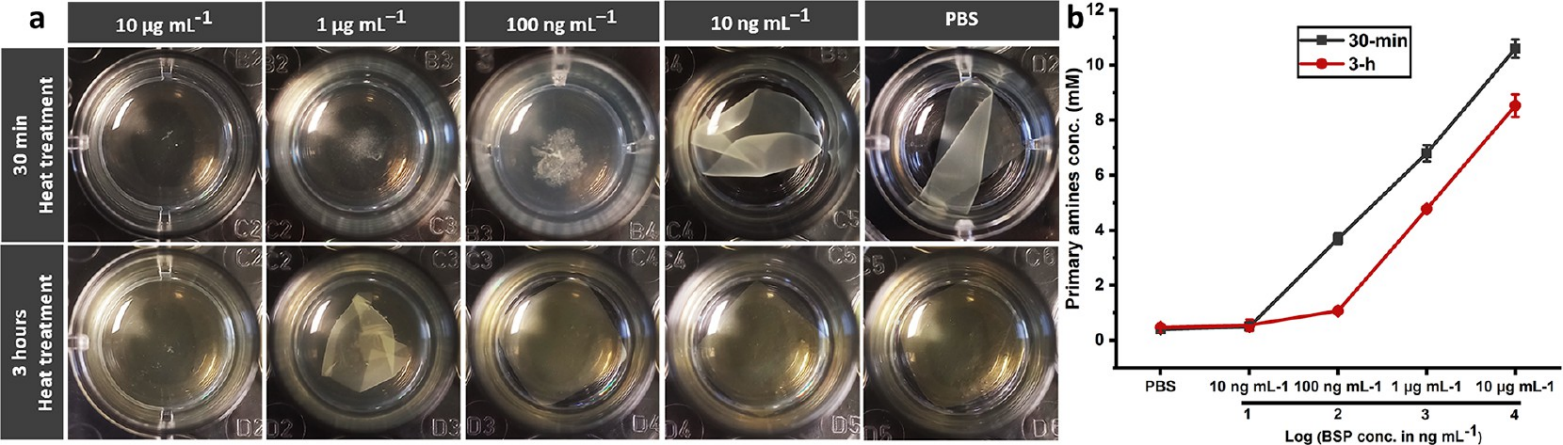
Influence of heat treatment duration on proteolytic degradation; (a) The state of the 30-min and 3-h samples after 1 day of incubation (37 °C, 150 rpm) in PBS containing BSP with various concentrations and in PBS; (b) Primary amines concentration in supernatants; error bars show the standard deviation; except for 3-h_1 μg mL^–1^, 3-h_blank of 10 ng mL^–1^, and 30 min_blank of the PBS group that averages of 2 measurements have been reported, all other cases are averages of 3 measurements. Graphs for 30-min samples in the range of 10 ng mL^–1^ to 10 μg mL^–1^ and for 3-h samples in the range of 100 ng mL^–1^ to 10 μg mL^–1^ are highly linear with regression coefficients of higher than 0.998.

The stability of 30-min and 3-h specimens was investigated in PBS containing BSP with different concentrations. The difference in susceptibility to proteolytic hydrolysis became evident in the early hours after incubation; 30-min heat-treated specimens were entirely disintegrated in less than 3 h at 10 and 1 μg mL^–1^ concentration levels of BSP, while 3-h counterparts still retained their integrity (Figure S2). After 1 day, the samples’ state was appraised visually and quantitatively by determining the primary amine concentration in supernatants. At 10 μg mL^–1^ concentration, both 30-min heat-treated and 3-h heat-treated specimens have disintegrated (Figure 6a); the concentration of primary amines in the 30-min heat-treated group was 24% higher than that in the 3-h group (Figure 6b). At 1 μg mL^–1^, 3-h samples still retained their integrity, while 30-min counterparts were nearly entirely solubilized (Figure 6a); so, the difference between primary amines content in 30-min heat-treated and 3-h heat-treated groups is expected to be to a larger extent compared to 10 μg mL^–1^ group and quantitative data support this speculation (42% difference; Figure 6b). At 100 ng mL^–1^, 30-min samples have entirely lost their integrity, but in the 3-h heat-treated group, there is no notable sign of deterioration (Figure 6a). These visual results match the primary amines’ content very well, which was around 3.5 times higher for the 30-min group compared to the 3-h group (Figure 6b). In 10 ng mL^–1^ and PBS groups, both 30-min heat-treated and 3-h heat-treated groups have retained their integrity (Figure 6a), and it could be postulated that there would not be too much difference in the number of primary amines; quantitative data has borne this out (Figure 6b). As can be seen in Figure 6b, interestingly, a highly linear correlation between the primary amines content of supernatants and the logarithm of BSP concentration in the base 10 was observed (for 30-min heat-treated samples in the range of 10 ng mL^–1^ to 10 μg mL^–1^, and for 3-h heat-treated samples, in the domain of 100 ng mL^–1^ to 10 μg mL^–1^).

**Figure 7:**
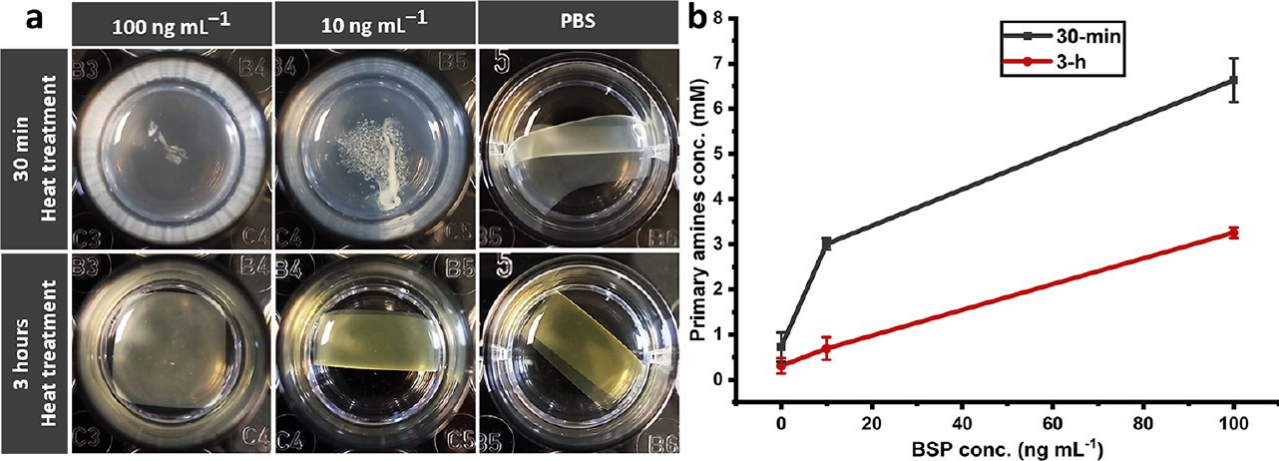
Influence of heat treatment duration on proteolytic degradation; the state of the 30-min and 3-h samples after 5 days of incubation (37 °C, 150 rpm) in PBS containing BSP at 100 and 10 ng mL^–1^ levels and in PBS. (a) Visual appearance. (b) Primary amine content (error bars show the standard deviation; data are the average of 2 measurements).

The state of the samples after incubation for 5 days was also assessed. Interestingly, even at this point, 3-h heat-treated samples kept their integrity in 100 and 10 ng mL^–1^ concentrations of BSP, while 30-min heat-treated counterparts were disintegrated even in 10 ng mL^–1^ concentration (Figure 7a); primary amine content is also remarkably higher in the supernatants of 30-min heat-treated samples in 100 and 10 ng mL^–1^ concentrations of BSP (Figure 7b). These observations further highlight the capacity of the heat treatment duration for modulation of proteolytic degradation.

It can be concluded that heat treatment is a feasible approach for making glycerin-containing casein-based material water-insoluble, and mechanical properties, water absorption capability, and hydrolytic stability could be easily tuned by the heat treatment duration. Apart from these conclusions, it was observed that 30-min heat-treated samples responded to BSP at concentrations as low as 100 ng mL^–1^; given its higher sensitivity compared to 3-h heat-treated counterparts, this group was deployed in the subsequent experiments.

### 3.6 Hydrolysis and Stability of the Selected Casein-Based Structure in Exposure to PPE

**Figure 8:**
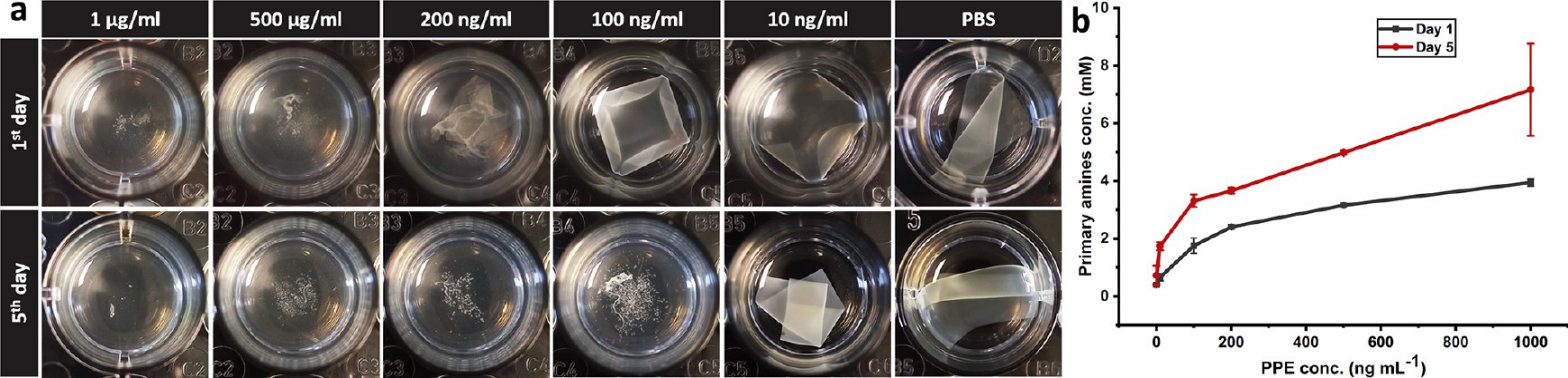
(a) Visual state of samples incubated in PBS containing PPE with different concentrations and in PBS (control) after 1 and 5 days. (b) Primary amine content in the supernatants (error bars show the standard deviation; data are the average of 2 measurements).

After encouraging results with BSP were obtained in terms of the sensitivity of 30-min heat-treated samples, their proteolytic stability in exposure to PPE as an elastase was also investigated. As mentioned earlier, PPE has been used as a model of the human leukocyte elastase,^32^ which is one of the key players in the inflammatory response. In experiments done with PPE with concentrations of 1 μg mL^–1^, 500 ng mL^–1^, and 200 ng mL^–1^ (in PBS), the samples lost their integrity in a maximum of 1 day (Figure 8a). After 5 days, specimens exposed to PPE with 100 ng mL^–1^ concentration also disintegrated, but at a concentration of 10 ng mL^–1^ and PBS, they still retained their integrity (Figure 8a). These data show that 30-min heat-treated specimens could respond to the PPE at concentrations as low as 100 ng mL^–1^ and serve for its detection. Quantification of the media’s primary amine content reveals an escalating tendency that is dependent on both time and PPE concentration (Figure 8b).

### 3.7 Hydrolysis and Stability of the Casein-Based Structure in Exposure to HNE

**Figure 9:**
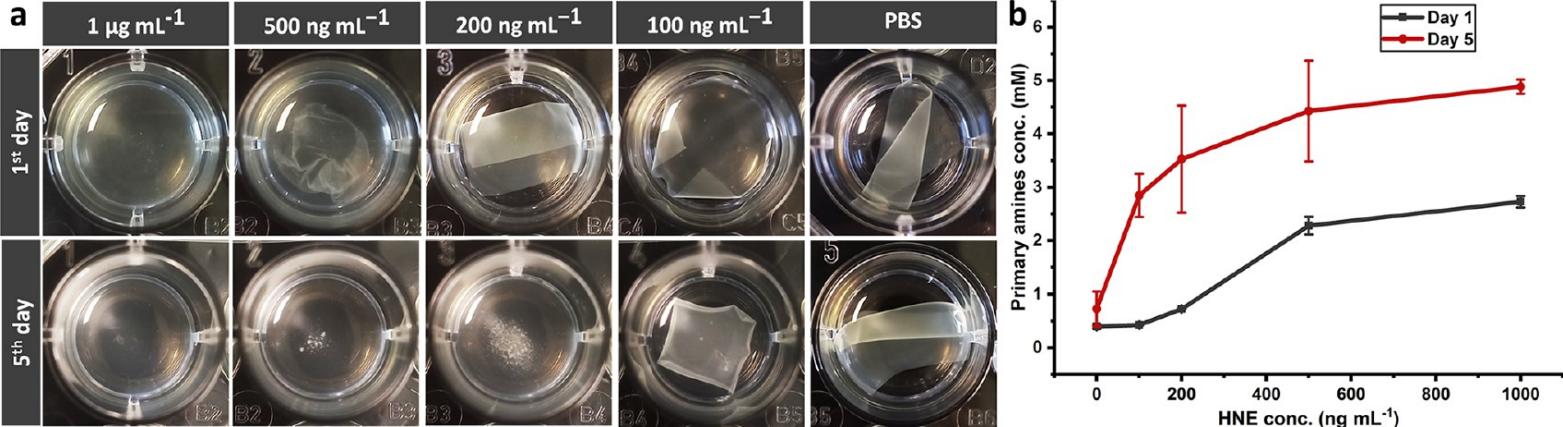
(a) The visual state of samples incubated in PBS containing HNE with various concentrations and in PBS (control) after 1 and 5 days; (b) Primary amine content in the supernatants (error bars show the standard deviation; data are the average of 2 measurements).

In the next step, the degradation of 30-min heat-treated films in exposure to HNE, one of the proteases generated by macrophages, was appraised. It is the protease that has been shown to be responsible for the degradation of fibronectin.^52^ In HNE, at 1 μg mL^–1^ (6.04 U mL^–1^) concentration, complete solubilization, and at 500 ng mL^–1^ (3.02 U mL^–1^), partial solubilization occurred on the first day (Figure 9a). After 5 days, samples at 200 ng mL^–1^ (1.21 U mL^–1^) were also disintegrated (Figure 9a). Primary amine quantification of supernatants at both time points revealed a dose-dependent increasing trend with an increase in the HNE concentration (Figure 9b). HNE activity in infected wounds’ exudates has been reported to be 22.97 ± 13.27 U mL^–1^.^12^ In another research, in 3 out of 4 investigated chronic wounds, an HNE concentration of higher than 2.5 μg mL^–1^, and in one of them, around 1 μg mL^–1^ was reported.^53^ The HNE activity and concentration reported in both studies are significantly larger than the levels to which the developed structure in the present research responded. Although our results are promising, any definite conclusion could be drawn only after clinical trials. Other researchers have also tried to develop some sensors for detecting HNE. They are mainly colorimetric; Yang and colleagues used filter and chromatography papers as the transducer and reported a sensitivity of 0.6 μg mL^–1^ in wound exudates.^53^ In our group, Hasmann et al. deployed modified silica gels to immobilize the HNE substrate, and based on spectrophotometric measurements, the developed material was able to distinguish infected and noninfected wounds.^12^ The approach followed in this study has some advantages compared to previous research. One of them is the lack of concern regarding the release of chromophore or fluorophore species with cytotoxic effects on the wound. The second benefit is that it is not dependent on the availability of a spectrophotometer or any other equipment or kit, and the proteolytic state of the wound can be tracked by the naked eye. The third advantage is that the interpretation of observations does not necessarily require special expertise. Therefore, observations could be assimilated by patients or the people who care for them after receiving an introduction. In addition, pictures can be taken and sent to medical professionals to assess the situation and decide what therapies to administer. In this way, unnecessary referrals to clinics could be reduced, and as a result, patient satisfaction could be improved. Last but not least, the cost-effectiveness of raw materials and the fabrication process make the product affordable for a broader range of patients, and it will alleviate the financial pressure on patients, healthcare systems, or insurance providers; this is especially of paramount importance for nations with lower per capita incomes.

### 3.8 Proteolytic activity of hydrolysates

**Figure 10:**
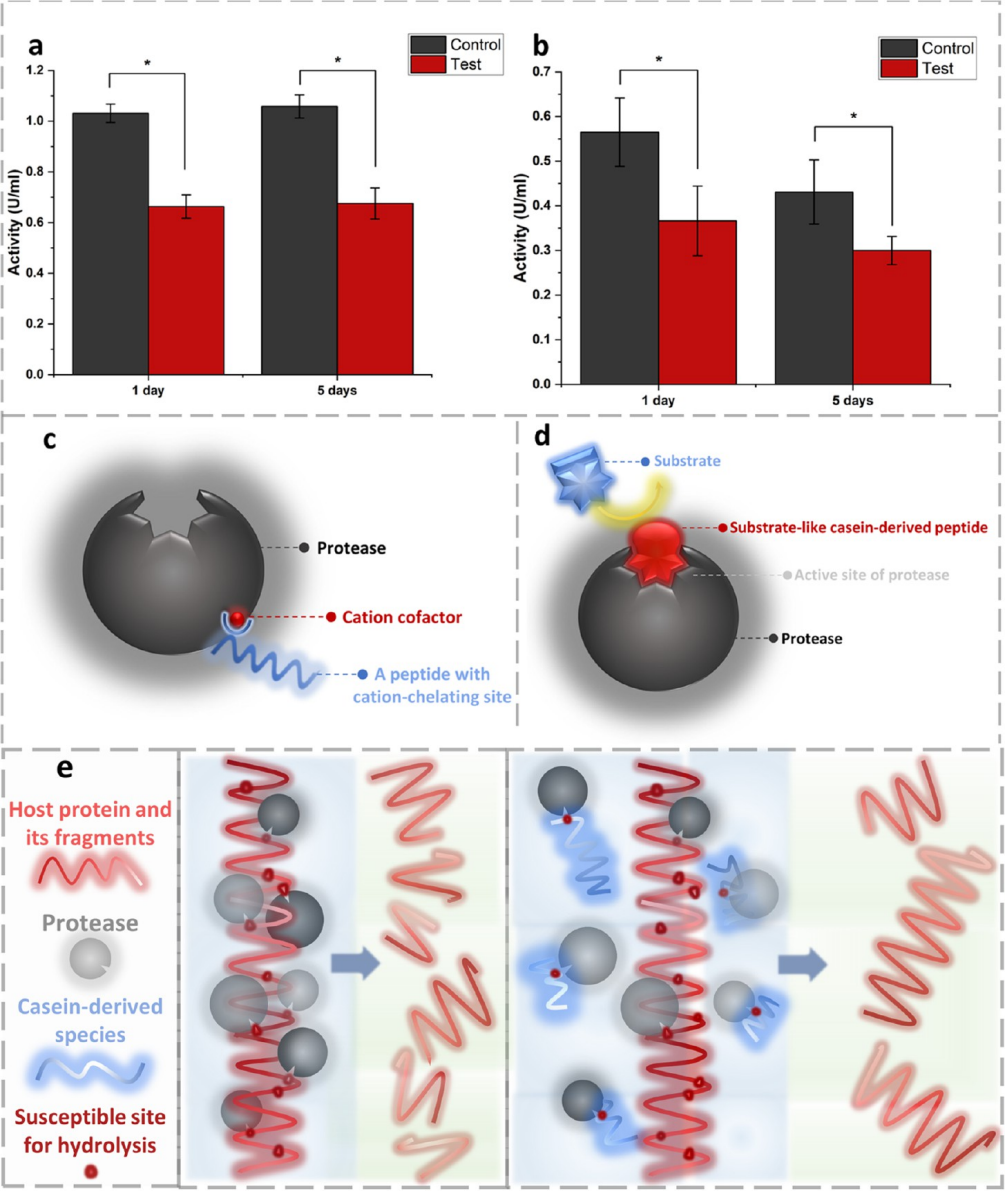
Protease activity in the supernatants resulting from hydrolysis of the casein films was compared to the activity of protease solutions incubated under identical conditions but in the absence of films (control) for (a) BSP and (b) PPE. Data are the average of at least 5 measurements. (c) Chelation of cationic cofactors of MMPs as a proposed explanation for the lower proteolytic activity of hydrolysates compared to the control (protease that had not been exposed to the casein-based samples). (d) Competitive inhibition is another potential reason for the lower proteolytic activity in the test group compared to the control group. (e) A comparison between the level of destruction of host proteins by proteases in the absence (the middle box) and the presence of casein and its fragments (the right box).

As mentioned in the introduction section, dysregulated proteolytic activity in chronic/infected wounds could be one of the deteriorating factors that hamper the normal healing process,^10^ so management of protease activity could be a judicious route for promoting wound healing. In the current research, to explore the potential of developed casein-based structures in attenuating the proteolytic activity, they were exposed to the proteases’ solutions (in PBS), and after 1 and 5 days, the protease activity of the media was measured. The films were disintegrated entirely (solubilized) under the investigated conditions due to hydrolysis catalyzed by proteases. Protease solutions in PBS, without casein-based specimens, were also incubated under conditions identical to those of the test group, and their protease activity was compared to the test group. According to the assay results, as depicted in Figure 10a and b, protease activity in the test group had been 30 to 36% lower than in the control group. The decrease in the BSP activity could be explained through two pathways. One of them is the sequestration of metalloproteases’ cofactors, especially zinc, by casein hydrolysates (Figure 10c). This is based on the fact that metalloproteases could constitute a part of the proteases secreted by *Bacillus sp.* bacteria^54^ and the cation-chelating ability of casein.^55^ Another explanation for the observed inhibitory effect is competitive inhibition. It happens when the inhibitor is similar to the substrate and forms a complex with the active site of the enzyme and, as a consequence, disturbs the enzyme’s function (Figure 10d). The latter route is more likely to be the primary mechanism because a comparable extent of inhibition was observed for BSP and PPE, while PPE is a serine protease, which acts without cationic cofactors. Hsieh et al. demonstrated that casein hydrolysates, derived from the enzymatic digestion of casein by a plant-based protease (bromelain), exhibited an inhibitory effect on prolyl endopeptidase, a peptidase implicated in neurodegenerative diseases.^56^ The observed inhibitory effect on both BSP and PPE holds the promise that the developed casein-based material could also protect the proteins in the wound site from the destructive activity of proteases (Figure 10e).

### 3.9 Antioxidant Properties of Hydrolysates

**Figure 11:**
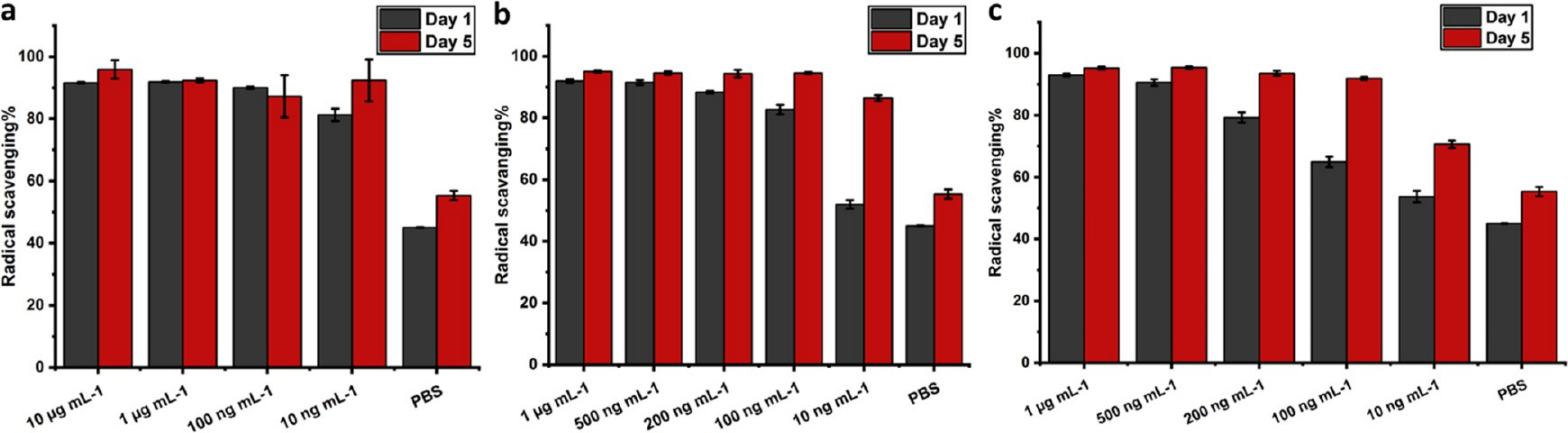
Radical scavenging potential of supernatants at different levels of (a) BSP, (b) PPE, and (c) HNE based on decolorization of ABTS cationic radicals (data are the average of at least 2 measurements).

Uncontrolled levels of reactive oxygen (and nitrogen) species could be another risk factor for impaired wound healing.^57^ In light of this, relieving oxidative stress and suppressing radical species have been widely considered as a strategy to promote healing. In the current study, supernatants of all groups showed a radical scavenging capability; 87 to 94% quenching of ABTS^•+^ was observed for hydrolysates of all 3 investigated proteases at 100 ng mL^–1^ concentration and higher at the fifth-day time-point (Figure 11). At 500 ng mL^–1^ and 1 μg mL^–1^ concentrations, at the first-day time-point, hydrolysates showed approximately 90% radical scavenging (Figure 11). Surprisingly, degradation products, even in the case of 10 ng mL^–1^ levels of BSP, PPE, HNE, and PBS groups, showed a considerable inhibitory effect; minimum 45% up to 92% at the first and fifth-day time points (Figure 11). A substantial increase of 15, 29, and 24% in the antioxidant effect upon hydrolysis progression from the first day to the fifth day in the HNE group at 200, 100, and 10 ng mL^–1^ concentrations is evident (Figure 11c).

In general, hydrogen and electron donation are the two main proposed action mechanisms of antioxidants,^58^ which, at least in part, could also be in charge of the observed antioxidant properties in this study. The antioxidant properties of casein hydrolysates have been of interest in many studies. One critical point that distinguishes current research from other studies is the idea of the in situ generation and release of hydrolysates with antioxidant properties instead of producing them separately and then using them. In-site liberation of bioactive peptides and polypeptides is supposed to also happen *in vivo* by hijacking pernicious proteases in chronic/infected wounds; the pure effect could be turning a threat (high levels of protease activity) into opportunities (release of casein fragments with antioxidant effect). Another distinguishing facet is the envisaged application; in almost all studies, food-related targets have been considered, such as preserving foods from damage due to oxidation, food packaging, or edible films. Although wound dressings with antioxidant properties have been fabricated by adding some agents to the dressing in numerous studies, to our knowledge, the concept that degradation products of a dressing provide species with an antioxidant effect has been less considered. More importantly, casein has not been deployed as a wound dressing with such a vision that it could benefit the wound concomitant with its in situ hydrolysis.

### 3.10 Antibacterial Test

No inhibition zone was observed in tests with both bacterial species (Figure S3), indicating that none was sensitive to the investigated hydrolysates. Some of the casein-derived peptides have antimicrobial properties.^59^ The fact that no antibacterial effect was observed in the present study could be due to the absence of antimicrobial peptides or their insufficient concentration in the hydrolysates.

### 3.11 *In-Vitro* Cell Culture Study

#### 3.11.1 Cell–Biomaterial Interaction

**Figure 12:**
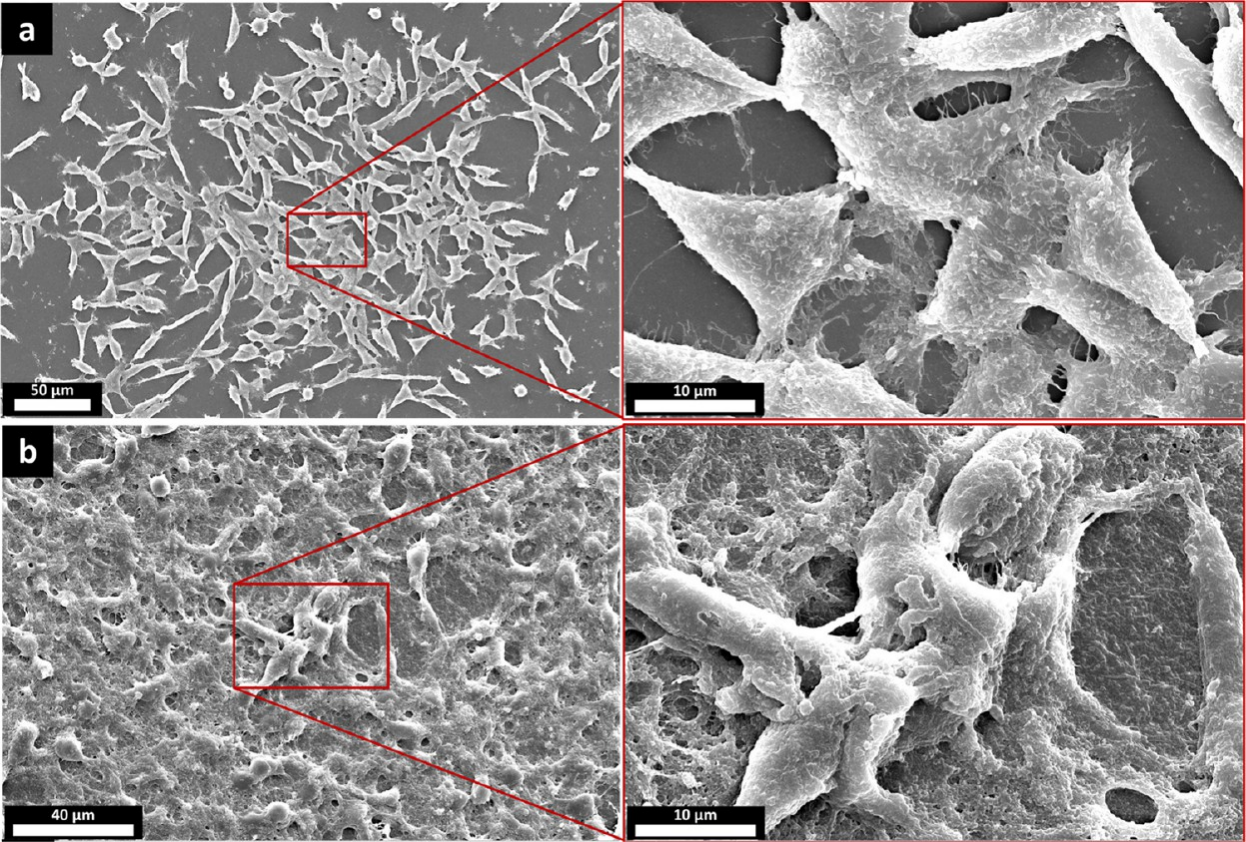
FE-SEM images from the samples, 5 days (a) and 7 days (b) post fibroblast culture; cells have attached, spread, proliferated, and expressed extracellular matrix over the surface of the casein-based structure.

Assessing the appearance of the cells cultured on the biomaterial’s surface provides insights into the biocompatibility of that material. Round-shaped cells usually indicate a lack of favorable cell–biomaterial interactions, whereas the extension of the cells over the surface is a sign of favorable interactions between the material and the cells. Most mammalian cells, including connective tissue cells, such as fibroblasts, are anchorage-dependent; this means that they need to adhere to a substrate to remain alive and functional. Adhesion affects the spreading, migration, proliferation, and differentiation of the cells^60^ and is of paramount importance in tissue engineering and regenerative medicine. Therefore, studying the cells’ appearance and attachment to the biomaterial is valuable. As described in Section 2.2.14.1, in this research, L929 fibroblasts were exposed to the developed casein-based membranes, and the morphology of the cells on the specimens was appraised by capturing FE-SEM images after 5 and 7 days. As is evident in Figure 12, cells were well elongated, spread over the surface, and developed extensions (filopodia). These micrographs indicate that the cells interacted suitably with both the samples’ surfaces and each other. Favorable interaction of cells with each other, in addition to favorable interaction with the biomaterial, is deemed to be essential for a suitable scaffold.^60^ On day 7, it seems that cells have covered the substrate (Figure 12b), which further supports the compatibility of the developed casein-based structure with fibroblasts. Interactions of the substrate with cells could be specific or nonspecific.^60^ Specific interaction means that cell receptors might recognize some particular domains in the substrate, and the cells attach to the substrate through them.^60^ Some peptides that are able to interact specifically with cell surface receptors have been identified.^61^ These peptides and their fragments were searched in the sequence of bovine αs_1_-, αs_2_-, β-, and K-casein, according to the UniProt database. While none of the cell-adhesion peptides (CAP) cited in ref (61) were exactly found in the casein molecules, some of them were very close to the sequences that exist in casein molecules. For instance, Isoleucine-Lysine-Leucine–Leucine-Isoleucine (IKLLI), Isoleucine-Aspartic acid-Alanine-Proline-Serine (IDAPS), Arginine-Glutamic acid-Aspartic acid-Valine (REDV), Leucine-Arginine-Glutamic acid (LRE), and Leucine-Aspartic acid-Valine (LDV) only differ in the first or last amino acid residue from the sequences in αs_1_-casein or β-casein. Some of the other CAPs that are partially encoded in the casein molecules are listed in Table S1. So, it could be speculated that maybe some sites in casein molecules interact with cell surface receptors. Another kind of interaction is nonspecific, which, unlike the specific interactions, is not receptor-dependent.^60^ Given that casein is made of various amino acids with different functional groups, it seems plausible that nonspecific interactions would also have contributed to the desirable interaction of the cells with the casein-based material. The fact that the developed material in this study supports the attachment of the cells is valuable. This characteristic is the Achilles heel of many biomaterials, both synthetic and natural, even those that are widely used in research, such as polydimethylsiloxane,^62^ aliphatic polyesters,^63^ poly(vinyl alcohol),^64^ or even biopolymers such as alginate,^65^ chitosan,^66^ and bacterial cellulose.^67^ As a result, in these cases, additional efforts are required to confer cell affinity to the material, for example, through blending,^68^ conjugation of CAP to molecule,^69^ or surface modification.^70^ To the best of our knowledge, this is the first time that cell interaction with a casein-based material has been studied in this manner.

#### 3.11.2 Evaluation of the Cytotoxicity of Hydrolysates

**Figure 13:**
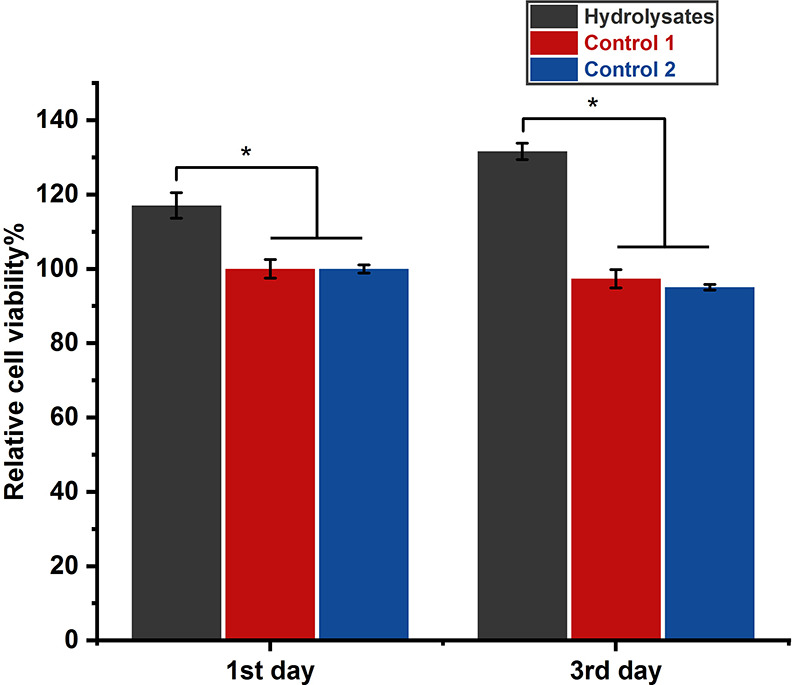
MTT assay of fibroblasts cultured in DMEM supplied with FBS (control 1), in a medium that was a 70:30 mixture of DMEM supplied with FBS and collagenase solution in PBS (control 2), or in a medium that was a 70:30 mixture of DMEM supplied with FBS and hydrolysate obtained from incubating the casein-based film in collagenase type I (500 μg mL^–1^) for 24 h (average of 3 data).

According to the MTT test, it seems that the hydrolysates not only did not cause any cytotoxicity but also increased cell proliferation by 30% in the culture medium (Figure 13). The collagenase solution in PBS, which was incubated under identical conditions and treated similarly to the hydrolysates, did not influence cell viability (control 2 in Figure 13). This finding would suggest that the hydrolysates’ beneficial effects are not attributable to the protease component that was along with them.

The hydrolysates may promote cell proliferation through two potential mechanisms. The first is supplying peptides and amino acids that can be taken up and metabolized by the cells, and the second is protecting the cells from oxidative stress.^71^ The latter hypothesis is consistent with the hydrolysates’ antioxidant properties discussed in Section 3.9.

## Conclusions

Casein-based films were made water-insoluble by heat treatment, a clean and straightforward approach. Heat treatment duration was proved to be a valuable tool for controlling the degradation rate, mechanical properties, and water absorption capability of casein-based films. The developed material could play multiple roles in wound management. First, it could serve as a scaffold if the proteolytic activity was low and did not cause its early disintegration. Second, it could indicate severe proteolytic activity by its disappearance or disintegration. Third, it could reduce the protease activity and eventually release antioxidant species during its degradation, which is expected to promote healing. Moreover, it could also contribute to moisture management.

## Supporting Information Available

FE-SEM images of the sample that was fractured after immersion in liquid nitrogen (Figure S1); the state of the samples heat treated for 2 different durations after 3 h of incubation in PBS containing BSP with 2 different concentrations (Figure S2); the results of the antibacterial test (Figure S3); cell adhesion peptides and some similar sequences in casein molecules (Table S1) (PDF)
