# Supplementary material for: Multifunctional Casein-Based Wound Dressing Capable of Monitoring and Moderating the Proteolytic Activity of Chronic Wounds
Source: Biomacromolecules. 2024 Jan 31;25(2):700–14. doi: 10.1021/acs.biomac.3c00910 (PMC10865360; doi:10.1021/acs.biomac.3c00910)
Supplement: Supplementary file 1 — bm3c00910_si_001.pdf [file bm3c00910_si_001.pdf]

A multifunctional casein-based wound dressing capable of monitoring and moderating the proteolytic activity of chronic wounds

Davood Kolahreez [1, 2, 3], Laleh Ghasemi-Mobarakeh [1§], Felice Quartinello [2], Falk Liebner [3], Georg M. Guebitz [2, 4], Doris Ribitsch [2, 4§*]*

[1] Department of Textile Engineering, Isfahan University of Technology, Isfahan 84156-83111, Iran

[2] Institute of Environmental Biotechnology, Department of Agrobiotechnology, IFA-Tulln, University of Natural Resources and Life Sciences, Vienna, Konrad-Lorenz-Strasse 20, 3430 Tulln an der Donau, Austria

[3] Institute of Chemistry of Renewable Resources, Department of Chemistry, University of Natural Resources and Life Sciences, Vienna, Konrad-Lorenz-Strasse 24, 3430 Tulln an der Donau, Austria

[4] Austrian Centre of Industrial Biotechnology (ACIB), Konrad-Lorenz-Strasse 20, 3430 Tulln an der Donau, Austria

* laleh.ghasemi@cc.iut.ac.ir

* doris.ribitsch@boku.ac.at

§ L.G. and D.R. contributed equally to this paper.

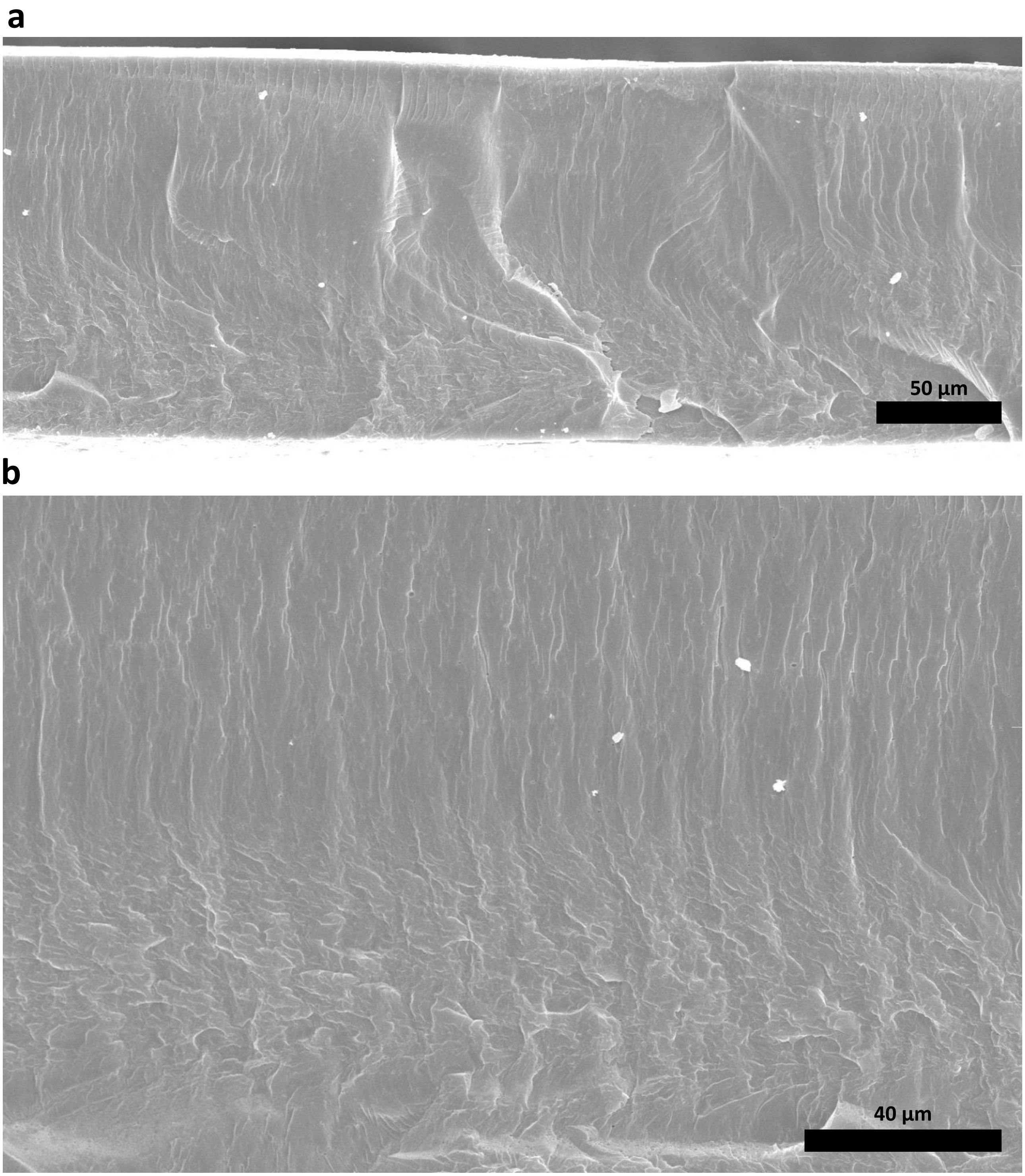

Figure S1. The FE-SEM images of the fractured cross-section of the glycerol-containing sample that was not heat treated; before fracture, the sample was immersed in liquid nitrogen

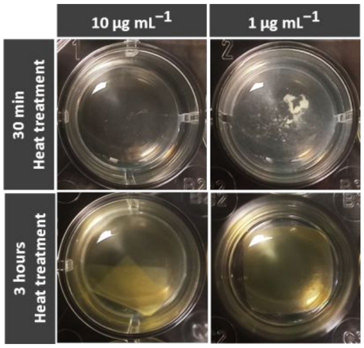

Figure S2. Influence of heat treatment duration on proteolytic degradation; the state of samples heat treated for 30 minutes and 3 hours after 3 hours of incubation (37 ˚C, 150 rpm) in PBS contained BSP with 10 μg mL^{-1} and 1 μg mL^{-1} concentrations

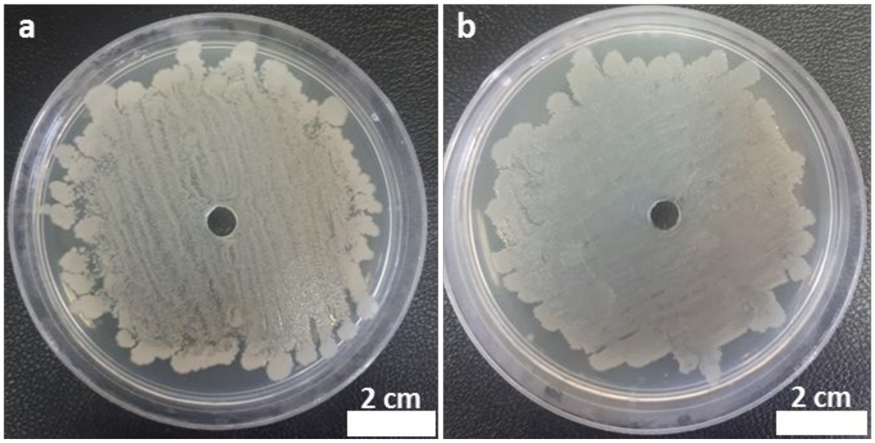

Figure S3. The results of the disc diffusion test for a) *Staphylococcus aureus* and b) *Escherichia coli* after 24 hours of incubation

Table S1. Cell adhesion peptides (adapted from reference [1]) and some similar sequences in casein molecules; the numbers in parenthesis show that the respective sequence has been repeated more than once.

Similar sequence in casein	Type of casein	CAP
**GE**	beta-casein	GFOGER<br>DGEA
**ER**	Alpha-S1-casein (2X)<br>Kappa-casein	GFOGER
**EA**	Alpha-S1-casein<br>Beta-casein<br>Kappa-casein (2X)	DGEA
**SR**	Kappa-casein	YIGSR<br>PDGSR<br>KRSR
**IGS**	Alpha-S1-casein (2X)<br>Alpha-S2-casein	YIGSR
**GS**	Alpha-S1-casein (2X)<br>Alpha-S2-casein	PDGSR

**YI**	Kappa-casein	YIGSR
**SI**	Alpha-S1-casein<br>Alpha-S2-casein (2x)<br>Beta-casein	SIKVAV<br>SINNNR<br>NPWHSIYITRFG
**VAV**	Alpha-S1-casein	SIKVAV
**AV**	Alpha-S1-casein<br>Alpha-S2-casein (2x)<br>Beta-casein<br>Kappa-casein (3X)	SIKVAV
**VA**	Alpha-S1-casein (3X)<br>Alpha-S2-casein (2x)<br>Beta-casein<br>Kappa-casein (2X)	SIKVAV
**KV**	Alpha-S1-casein (2X)<br>Alpha-S2-casein<br>Beta-casein (3X)	SIKVAV
**IK**	Alpha-S1-casein (2X)	SIKVAV
**KLLI**	Alpha-S1-casein	IKLLI<br>EIKLLIS [2]
**LR**	Alpha-S1-casein (2X)	LRGDN<br>LRE

		GTFALRGDNGQ<br>CFALRGDNP
**RG**	Beta-casein	LRGDN<br>GTFALRGDNGQ<br>GRGDS<br>PKRGDL
**NR**	Alpha-S2-casein	SINNNR
**NN**	Kappa-casein	SINNNR
**NN**	Kappa-casein	SINNNR
**INN**	Kappa-casein	SINNNR
**RE**	Alpha-S2-casein<br>Beta-casein	LRE
**GT**	Alpha-S1-casein	GTFALRGDNGQ
**NP**	Alpha-S1-casein<br>Alpha-S2-casein (2X)	CFALRGDNP
**NPW**	Alpha-S2-casein	NPWHSIYITRFG
**FG**	Alpha-S1-casein	NPWHSIYITRFG
**YI**	Kappa-casein	NPWHSIYITRFG
**RF**	Alpha-S1-casein<br>Kappa-casein	NPWHSIYITRFG
**ITR**	Beta-casein	NPWHSIYITRFG

**IT**	Alpha-S2-casein (2X)<br>Beta-casein	NPWHSIYITRFG
**HS**	Alpha-S1-casein	NPWHSIYITRFG
**RN**	Alpha-S2-casein	TWYKIAFQRNRK
**QR**	Alpha-S2-casein<br>Beta-casein	TWYKIAFQRNRK
**FQ**	Beta-casein	TWYKIAFQRNRK
**AF**	Beta-casein	TWYKIAFQRNRK
**KIA**	Kappa-casein	TWYKIAFQRNRK
**KI**	Alpha-S2-casein (2X)<br>Beta-casein (2X)<br>Kappa-casein	TWYKIAFQRNRK
**YK**	Alpha-S1-casein<br>Alpha-S2-casein	TWYKIAFQRNRK
**WY**	Alpha-S1-casein	TWYKIAFQRNRK
**LGT**	Alpha-S1-casein	LGTIPG
**LG**	Alpha-S1-casein (2X)<br>Beta-casein<br>Kappa-casein	LGTIPG
**PG**	Beta-casein	LGTIPG
**IP**	Alpha-S1-casein	LGTIPG

	Alpha-S2-casein<br>Beta-casein (2x)<br>Kappa-casein (3X)	
**TI**	Kappa-casein (4X)	LGTIPG
**PK**	Alpha-S1-casein<br>Alpha-S2-casein<br>Beta-casein (2x)<br>Kappa-casein	PKRGDL
**DAPS**	Alpha-S1-casein	IDAPS
**PS**	Alpha-S1-casein (3X)<br>Alpha-S2-casein<br>Kappa-casein	IDAPS
**DAP**	Alpha-S1-casein	IDAPS<br>KLDAPT
**DA**	Alpha-S1-casein (2X)	IDAPS
**EDV**	Alpha-S1-casein	REDV
**PH**	Beta-casein<br>Kappa-casein (2x)	PHSRN
**DV**	Alpha-S1-casein<br>Beta-casein	LDV<br>AELDVP
**LD**	Alpha-S1-casein	LDV

**PP**	Beta-casein (4X)<br>Kappa-casein (2x)	WQPPRARI
**SPP**	Kappa-casein	SPPRRARV
**LDA**	Alpha-S1-casein	KLDAPT
**YGL**	Kappa-casein	SVVYGLR
**VPG**	Beta-casein	VPGIG
**VAP**	Alpha-S1-casein	VGVAPG
**NYY**	Kappa-casein	MNYYSNS<br>CNYYSNS
**DVP**	Alpha-S1-casein	AELDVP
**VAL**	Alpha-S1-casein<br>Alpha-S2-casein<br>Beta-casein<br>Kappa-casein	VALDEP
**SIG**	Alpha-S2-casein	SIGFRGDGQTC

REFERENCES

(1) Huettner, N.; Dargaville, T. R.; Forget, A. Discovering Cell-Adhesion Peptides in Tissue Engineering: Beyond RGD. *Trends Biotechnol.* **2018**, *36* (4), 372–383.

DOI:10.1016/j.tibtech.2018.01.008.

(2) TASHIRO, K.; MONJI, A.; YOSHIDA, I.; HAYASHI, Y.; MATSUDA, K.; TASHIRO, N.; MITSUYAMA, Y. An IKLLI-Containing Peptide Derived from the Laminin Α1 Chain Mediating Heparin-Binding, Cell Adhesion, Neurite Outgrowth and Proliferation, Represents a Binding Site for Integrin Α3β1 and Heparan Sulphate Proteoglycan. *Biochem. J.* **1999**, *340* (1), 119–126. DOI:10.1042/bj3400119.
